# New Drugs, Appliances, and Things Medical

**Published:** 1891-08-29

**Authors:** 


### NEW DRUGS, APPLIANCES, AND THINGS MEDICAL.

[All preparations, appliances, novelties, etc., of which a notice is desired, should be sent for The Editor, to care of The Manager, 140, Strand, London, W.C.]

### AT THE BRITISH MEDICAL ASSOCIATION.

Section A.—Food Stuffs and Drugs.

Meat Preparations.—First table. We noticed the J. B. Brush Manufacturing Company, 32, Snow Hill, London, with their fluid beef solution, Bovinine, which contains 20 per cent. of soluble albuminoids. This and the next preparation we have recently favourably noticed in our columns.

Liquor Carnis Company, 50, Holborn Viaduct. Besides the fluid Liquor Carnis, we noticed Caffyn's Carnis Suppositories. These are the liq. carnis plus a colloid medium, and for rectal feeding. *Jelly carnis*, or concentrated beef tea. This is a preparation of liquor containing 66 per cent., and is practically good strong beef tea, plus the soluble albuminoids. *Malto carnis.* This contains two-thirds of the liquor, with extract of malt and cocoa, and forms a nutritious and stimulating food for invalids and infants.

Messrs. Tucker, 51, Paddington Street, W. This stall exhibited the finest varieties of Eucalyptus preparations, and other Australian products, such as red gum. Some time back we noticed at length Mr. Curgenven's paper on the antiseptic results obtained by employing Messrs. Tucker's preparations of eucalyptus in scarlet fever and diphtheria.

Henri Nestlé, 9, Snow Hill, E.C.—The exhibit of this famous food for infants was well patronised. By-the-bye, the food consists of the finest baked wheat flour and concentrated milk. It forms a very nutritious, digestible powder, which has the advantage of being able to be kept for a very long time and of travelling without becoming deteriorated. Baking makes the flour soluble by breaking up the starch granules and partial transformation of them into dextrin, whilst the albuminoids are rendered soluble.

J. F. Macfarlane and Co., 17, North Bridge, Edinburgh, and 71, Coleman Street, London.—An excellent exhibit of the various antiseptic surgical appliances in the way of wools, gauges, bandages, ligatures, &c., made after the approved formulæ of Sir Joseph Lister and others.

G. Mellin, Marlborough Works, Peckham, London, S.E.—Here, again, we have a series of foods based on the original Mellin's food. The food is a brownish granular powder prepared from cereal grains, in which the starch has been converted into its soluble products by the action of vegetable diastase. From it are made Lacto-glycose. The food, plus fresh cow's milk, Mellin's Food biscuits, 58 per cent. of the food with wheaten flour. Adapted for children and others, to whom an entirely soluble food is not suitable. Mellin's cod-liver oil emulsion contains 50 per cent. of the finest Norwegian oil, and is prepared by trituration with solution of gum arabic and sugar. No saponification results, and consequently there is no alteration of the oil. The emulsion is prepared with or without hydrophosphates. Vin de Coca "Mellin" (unsweetened), made with simple red wine, and so less likely to cause the ill-effects of a sweet wine, and also the tonic effects are more easily produced.

Next we come to the combined exhibit of Carnrick & Co. with the Maltine Manufacturing Co. (Limited), Hart Street, Bloomsbury. There is a good collection of the various predigested foods of the former (peptonoids) and the maltine preparations of the latter, also specimens of the "Victoria" Ofner bitter water, which is particularly rich in the sulphates of soda and magnesia.

Packham and Co., Croydon. had an excellent stall, exhibiting their effervescing waters, which included many others than the Standard B. P. preparations.

The New York Pharmaceutical Co., 47, Farringdon Street, exhibited their gynœcological remedy, "Hayden's Viburnum Compound."

Thomas Christy and Co., 25, Lime Street, E.C., had a most interesting table. We always expect to find something new whenever this firm attends a Congress, and we seemed to have more than ever, including many surgical appliances as well. There were the preparations of the Burra Gookeroo seed which has well marked diuretic tonic and aphrodisiac properties. Pichi preparations, stated to be very successful for the nocturnal incontinence of urine of childhood. Jatamansi root, which is supposed to be the real Sumbul root and to be of very considerable power in epilepsy, and the various dressings made from the Fibrine Christia which is described as water, oil and spirit proof.

Feltoe and Smith, 27, Albemarle Street, W., had a display of their well-known "Specialité," Lime Juice Cordial. It is described as more concentrated than most preparations of this juice, and so more economical in its use.

Chemists' Aerated and Mineral Water Association made an interesting exhibit of their manufactures, showing the materials from which they are made, and the methods in which they are put up.

C. J. Hewlett and Son, Charlotte Street, E.C., showed excellent specimens of their concentrated mixtures, made from selected formulæ of their own and the pharmacopœias of the various London hospitals. Also compound liquors, coated pills, suppositories, antiseptic jelly, forming a very neat and tasteful exhibit.

Loeflund's Dietetic Malt and Malt Products (Robert Baelz and Co., St. Mary's Chambers, St. Mary Axe, E.C.).—These are made from the finest malt and Alpine milk. The Cremor Hordeatus has been found of great use in cases where cod-liver oil could not be taken.

Then come Messrs. Riddle with their Stower's Lime Juice Preparations. This old friend makes a famous summer drink. We notice her Majesty indulges in it.

Burgoyne, Burbidges, and Co., Coleman Street, E.C., had a stall on which many American firms were represented, such as Messrs. Park, Davis, and Co., Sultan Drug Co. (manufacturers of cactina, for heart cases), Rio Chemical Company, who produce Celerina, Aletris Cordial, and the like, and G. H. Kennedy's Pinus Canadensis.

Sanitas Company (Limited), Letchford's Buildings, Bethnal Green, E. This well-known company had a large show of their sanitary productions. In fact, the air in the vicinity of their table was largely perfumed with the volatile emanations of the "Sanitas" group. The automatic disinfector was favourably noticed, as well as Kingsett's peroxide of hydrogen preparations. The mercuric bactericide seems a great improvement on simple perchloride solution, as it does not coagulate the albuminous substances which are always present in operations. We believe that dentists find this a first-rate antiseptic fluid to wash out cavities with. "Sanitas" was also exhibited in the forms of jelly, soap, powder, gauze wool, and the thousand and one forms in which modern antiseptic substances are constructed for the various uses they are put to.

Apollinaris Company, Regent Street, W., had a pretty exhibit of their three excellent waters, Apollinaris, Hungarian Aperient (Diamond mark), and Friedrichshall.

Oppenheimer and Co., Sun Street, Finsbury Square, had a very ingenious invention on view, called Palatinoids. These are flat capsules made with acacia and sugar, by which drugs are administered free without any excipient whatever. Some are so formed that the ingredients in them may be kept separated until set free in the stomach, when the double decomposition desired is set up, such as is met with in Blaud's pill, for instance; the dosage is claimed to be extremely accurate. There were also excellent specimens of different "Liquors," such as Caulophyllin et Pulsatillæ co. and Euonymus, with pepsin, bismuth, &c.

B. Kühn, 36, St. Mary-at-Hill, Eastcheap, E.C., had an interesting exhibit of the new digestive ferment, papain, showing the fruit of the Carica Papaya, its juice, and the ferment. Also preparations of the latter, such as pills, lozenges, and liquor, acid glycerine, and papain. Also a series of albuminoids resulting from the papain digestion forming peptone foods. These either alone or combined with chocolate and cocoa. It is worth notice that the ferment is stated to be more active in neutral or slighly alkaline fluids.

M. Hoff, 29, New Bridge Street, E.C., showed excellent and varied forms of Hamburg Hoff's Malt Extract, and two new beverages, "the Meteor Beer," ordinary beer freed from alcohol by distillation, and Malto selzine, a non-intoxicating lemonade, of considerable food value.

Hertz and Collingwood, 4, Sussex Place, Leadenhall Street, E.C., showed "Franz Joseph" natural aperient water, a water containing a very large amount of the sodium and magnesium sulphate, with but slight mineral flavour. A very fine cod-liver oil, which is expressed on board the boats immediately from the livers, so that there is not the slightest chance of taint attaching to the oil. Levico Natural Arsenico Ferric Water: This we gave an account of some time back in our columns. We still use it largely, and with increasing confidence. The Sussex Patent Iodofume and Bromofume night lights: These give off the vapours of iodine and bromine when lighted, and are excellent for disinfection and deodorisation.

Geo. Mason and Co. (Limited), King's Road, Chelsea, manufacturers of specialities for invalids, had a very interesting collection of their manufactures, including essence of beef, concentrated beef tea, and O. K. Bouillon. This makes splended beef tea (we noticed this some time back with approbation). Malted food for infants, particularly remarkable for preserving the taste of the cereals and absence of bitterness. Meat lozenges, a very portable and convenient form of concentrated nourishment.

Willows, Francis, and Butler, 101, High Holborn, W.C., had along series of first-rate pharmaceutical liquors. We noticed more particularly their new liquor Calcis Iodinatæ. This is an extremely powerful preparation of iodine and lime, and has produced very good results in rheumatism, syphilis, and chronic affections of the lungs and stomach. Externally it is a good disinfectant, deodorant, and parasiticide.

Next we come to the well-stocked exhibit of a well-known firm, Brand and Co., Little Stanhope Street, Mayfair. Who does not know of Brand's essence of beef and chicken, his meat lozenges, and his invalid turtle soup? Besides these there are soups of all kinds, digestive meat biscuits, calf's foot jelly, lemon and orange jellies, and the predigested peptones of beef, mutton, veal, and chicken, all good, and what is more, palatable.

Coming to the end of the second table, we notice the Bouillon Fleet Co. (Limited), Camberwell, London, with their popular preparation Bouillon Fleet, or Fluid Beef Tea, &c.; also Kemmerick's Extract of Beef, prepared from lean beef only, and with this is Kemmerick's Peptone of Beef, especially made for invalids, and likewise the Bouillon Fleet Lozenges for travellers.

---

Those who have read "Looking Backward," can, by reading Mons. M. G. Molinari's book, see both sides of the question, and draw his own conclusions. The author of "Looking Backward" thinks that in another century society will be collective or on a socialistic basis, whereas Mons. Molinari, that the economic system will prevail. As we shall none of us be live in that time, Mons. Molinari and Mr. Bellamy can be left to decide the matter for themselves, without hurting any one's feelings but their own.

